# *cis*-[1,2-Bis(diphenyl­arsan­yl)ethane-κ^2^ *As*,*As*’]tetra­carbonyl­chromium(0)

**DOI:** 10.1107/S1600536811032314

**Published:** 2011-08-27

**Authors:** M. N. Norlidah, M. Y. Azhar, Omar Bin Shawkataly, Mohd Mustaqim Rosli, Hoong-Kun Fun

**Affiliations:** a Faculty of Industrial Science and Technology, Universiti Malaysia Pahang, Gambang 26300, Pahang, Malaysia; b Chemical Sciences Programme, School of Distance Education, Universiti Sains Malaysia, 11800 USM, Penang, Malaysia; c X-ray Crystallography Unit, School of Physics, Universiti Sains Malaysia, 11800 USM, Penang, Malaysia

## Abstract

In the title compound, [Cr(C_26_H_24_As_2_)(CO)_4_], the Cr atom is octa­hedrally coordinated by four carbonyl ligands and one bidentate 1,2-bis­(diphenyl­arsan­yl)ethane ligand, which chelates in a *cis* manner with an As—Cr—As bite angle of 82.513 (9)°. The dihedral angles between the pairs of benzene rings attached to each As atom are 84.63 (9) and 77.15 (8)°. In the crystal, mol­ecules are linked by C—H⋯O inter­actions, forming infinite chains along the *a* axis. The crystal structure is further stabilized by C—H⋯π inter­actions.

## Related literature

X-ray structure determinations of chromium carbonyls with arsine ligands are rare. A search of the Cambridge Crystallographic Structural Database (Allen, 2002 ▶) reveals only 12 complexes of chromium carbonyl disubtituted with tertiary arsines. For related structures, see: Bennett *et al.* (1971 ▶); Nowell *et al.* (1972 ▶). For the stability of the temperature controller used in the data collection, see: Cosier & Glazer (1986 ▶).
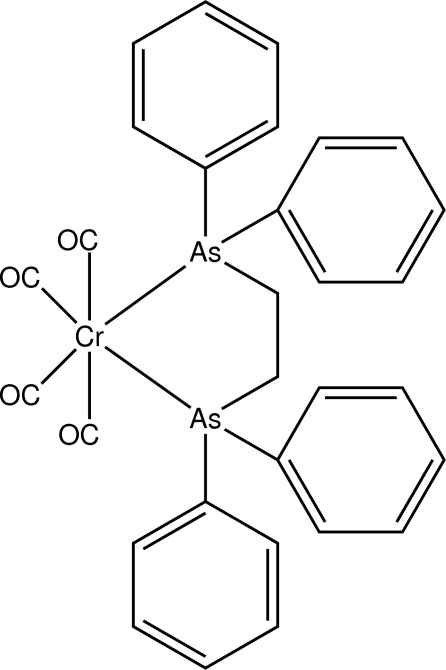


## Experimental

### 

#### Crystal data

[Cr(C_26_H_24_As_2_)(CO)_4_]
*M* *_r_* = 650.33
Orthorhombic, 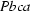

*a* = 17.0231 (4) Å
*b* = 12.6200 (3) Å
*c* = 25.5527 (6) Å
*V* = 5489.5 (2) Å^3^
*Z* = 8
Mo *K*α radiation
μ = 2.84 mm^−1^
*T* = 100 K
0.53 × 0.25 × 0.05 mm

#### Data collection

Bruker SMART APEXII CCD area-detector diffractometer
Absorption correction: multi-scan (*SADABS*; Bruker, 2009 ▶) *T*_min_ = 0.313, *T*_max_ = 0.871
87112 measured reflections
9352 independent reflections
7454 reflections with *I* > 2σ(*I*)
*R*_int_ = 0.054

#### Refinement

*R*[*F* ^2^ > 2σ(*F* ^2^)] = 0.028
*wR*(*F* ^2^) = 0.065
*S* = 1.02
9352 reflections
334 parameters
H-atom parameters constrained
Δρ_max_ = 0.78 e Å^−3^
Δρ_min_ = −0.50 e Å^−3^

### 

Data collection: *APEX2* (Bruker, 2009 ▶); cell refinement: *SAINT* (Bruker, 2009 ▶); data reduction: *SAINT*; program(s) used to solve structure: *SHELXTL* (Sheldrick, 2008 ▶); program(s) used to refine structure: *SHELXTL*; molecular graphics: *SHELXTL*; software used to prepare material for publication: *SHELXTL* and *PLATON* (Spek, 2009 ▶).

## Supplementary Material

Crystal structure: contains datablock(s) I, global. DOI: 10.1107/S1600536811032314/ng5210sup1.cif

Structure factors: contains datablock(s) I. DOI: 10.1107/S1600536811032314/ng5210Isup2.hkl

Additional supplementary materials:  crystallographic information; 3D view; checkCIF report

## Figures and Tables

**Table 1:** Selected bond lengths (Å)

As1—Cr1	2.4461 (3)
As2—Cr1	2.4512 (3)
Cr1—C2	1.8457 (17)
Cr1—C1	1.8511 (17)
Cr1—C3	1.8894 (17)
Cr1—C4	1.8935 (18)

**Table 2:** Hydrogen-bond geometry (Å, °) *Cg*1 is the centroid of the C7–C12 ring.

*D*—H⋯*A*	*D*—H	H⋯*A*	*D*⋯*A*	*D*—H⋯*A*
C9—H9*A*⋯O1^i^	0.93	2.57	3.345 (2)	141
C16—H16*A*⋯*Cg*1^ii^	0.93	2.60	3.519 (2)	169

Symmetry codes: (i) 
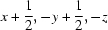
; (ii) 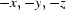.

**Fig. 1.**
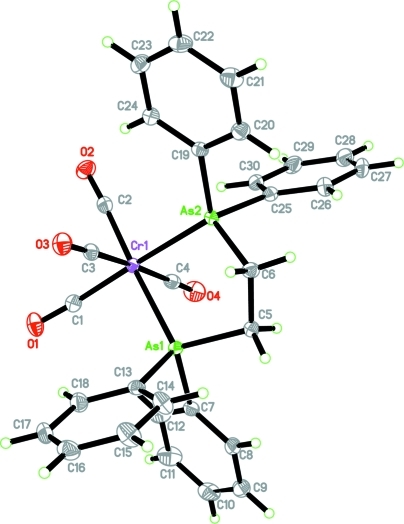
The molecular structure, showing 50% probability displacement ellipsoids. Hydrogen atoms are shown as spheres of arbitrary radius.

**Fig. 2.**
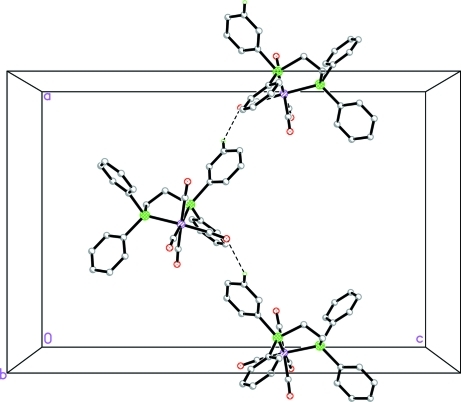
The packing of (I) shown an infinite chains along a axis. Dashed lines indicate hydrogen bonds. H atoms not involved in the hydrogen bond interactions have been omitted for clarity.

## supplementary crystallographic information

### Comment

Very few chromium carbonyls with bidentate arsine ligands have been reported (Allen, 2002). The C—C bond length and the As—Cr—As bite angle of the title complex are comparable to similar complexes of chromium carbonyls substituted with bidentate arsine with two carbon atom backbone (Nowell *et al.*,1972). The title compound is isostructural to Cr(CO)_4_(Ph_2_P(CH_2_)_2_PPh_2_) and this gives further support that the formation of stable *cis*-*M*(CO)_4_*L*_2_ compounds is prefered when the *L*_2_ groups are combined in a chelating bidentate ligand (Bennett *et al.* 1971).

The Cr—As bond lengths show an average value of 2.449 Å and the As—Cr—As bite angle has a value of 82.51 (1)° (Table 1) while in Cr(CO)_4_(Ph_2_P(CH_2_)_2_PPh_2_), the average of Cr—P bond lengths is 2.360Å and the P—Cr—P bite angle has a value of 83.41 (8).

In the molecule, the dihedral angle between the two benzene ring attached to the As1 and As2 are 84.63 (9)° (C7—C12 & C13—C18 rings) and 77.15 (8)° (C19—C24 & C25—C30 rings), respectively. The molecules form infinite chains along the *a* axis (Fig. 2) throught C9—H9A···O1^i^ (Table 2) intermolecular interactions. The crystal structure is further stabilized by C—H···π interaction involving *Cg*1, *Cg*1 is the centroid of C7—C12 (Table 2).

### Experimental

All manipulations were performed under a dry, oxygen-free nitrogen atmosphere using standard Schlenk techniques. All solvents were dried over sodium under dry oxygen free nitrogen. Chromium hexacarbonyl (200 mg, 0.909 mmol) and ethylenebisdiphenyl-arsanylethane (441.9 mg, 0.9086 mmol) in 35 ml of pet ether (100–130°C) was refluxed for 12 h. Suitable single crystals were obtained by solvent-solvent diffusion in a mixture of dichloromethane/methanol.

### Refinement

All hydrogen atoms were positioned geometrically and refined using ariding model with C—H = 0.93–0.97Å and *U*_iso_(H) = 1.2*U*_eq_(C).

### Crystal data

**Table:**

[Cr(C_26_H_24_As_2_)(CO)_4_]	*F*(000) = 2608
*M**_r_* = 650.33	*D*_x_ = 1.574 Mg m^−^^3^
Orthorhombic, *P**b**c**a*	Mo *K*α radiation, λ = 0.71073 Å
Hall symbol: -P 2ac 2ab	Cell parameters from 9793 reflections
*a* = 17.0231 (4) Å	θ = 2.4–31.6°
*b* = 12.6200 (3) Å	µ = 2.84 mm^−^^1^
*c* = 25.5527 (6) Å	*T* = 100 K
*V* = 5489.5 (2) Å^3^	Plate, yellow
*Z* = 8	0.53 × 0.25 × 0.05 mm

### Data collection

**Table:**

Bruker SMART APEXII CCD area-detector diffractometer	9352 independent reflections
Radiation source: fine-focus sealed tube	7454 reflections with *I* > 2σ(*I*)
graphite	*R*_int_ = 0.054
φ and ω scans	θ_max_ = 31.9°, θ_min_ = 2.0°
Absorption correction: multi-scan (*SADABS*; Bruker, 2009)	*h* = −25→25
*T*_min_ = 0.313, *T*_max_ = 0.871	*k* = −18→18
87112 measured reflections	*l* = −37→36

### Refinement

**Table:**

Refinement on *F*^2^	Primary atom site location: structure-invariant direct methods
Least-squares matrix: full	Secondary atom site location: difference Fourier map
*R*[*F*^2^ > 2σ(*F*^2^)] = 0.028	Hydrogen site location: inferred from neighbouring sites
*wR*(*F*^2^) = 0.065	H-atom parameters constrained
*S* = 1.02	*w* = 1/[σ^2^(*F*_o_^2^) + (0.0273*P*)^2^ + 3.1824*P*] where *P* = (*F*_o_^2^ + 2*F*_c_^2^)/3
9352 reflections	(Δ/σ)_max_ = 0.001
334 parameters	Δρ_max_ = 0.78 e Å^−^^3^
0 restraints	Δρ_min_ = −0.50 e Å^−^^3^

### Special details

**Table:**

Experimental. The crystal was placed in the cold stream of an Oxford Cryosystems Cobra open-flow nitrogen cryostat (Cosier & Glazer, 1986) operating at 100.0 (1) K.
Geometry. All e.s.d.'s (except the e.s.d. in the dihedral angle between two l.s. planes) are estimated using the full covariance matrix. The cell e.s.d.'s are taken into account individually in the estimation of e.s.d.'s in distances, angles and torsion angles; correlations between e.s.d.'s in cell parameters are only used when they are defined by crystal symmetry. An approximate (isotropic) treatment of cell e.s.d.'s is used for estimating e.s.d.'s involving l.s. planes.
Refinement. Refinement of *F*^2^ against ALL reflections. The weighted *R*-factor *wR* and goodness of fit *S* are based on *F*^2^, conventional *R*-factors *R* are based on *F*, with *F* set to zero for negative *F*^2^. The threshold expression of *F*^2^ > σ(*F*^2^) is used only for calculating *R*-factors(gt) *etc*. and is not relevant to the choice of reflections for refinement. *R*-factors based on *F*^2^ are statistically about twice as large as those based on *F*, and *R*- factors based on ALL data will be even larger.

### Fractional atomic coordinates and isotropic or equivalent isotropic displacement parameters (Å^2^)

**Table:**

	*x*	*y*	*z*	*U*_iso_*/*U*_eq_	
As1	0.057670 (9)	0.238802 (12)	0.109865 (6)	0.01225 (4)	
As2	0.018356 (9)	0.355446 (12)	0.219264 (6)	0.01278 (4)	
Cr1	−0.014160 (15)	0.402272 (19)	0.128720 (10)	0.01236 (5)	
O1	−0.06677 (8)	0.43384 (10)	0.01759 (5)	0.0246 (3)	
O2	−0.09528 (8)	0.60856 (9)	0.15170 (5)	0.0232 (3)	
O3	−0.16549 (7)	0.27784 (10)	0.14160 (5)	0.0224 (3)	
O4	0.14071 (8)	0.51681 (11)	0.11081 (6)	0.0272 (3)	
C1	−0.04384 (10)	0.42144 (12)	0.05967 (7)	0.0165 (3)	
C2	−0.06388 (10)	0.52821 (13)	0.14459 (7)	0.0169 (3)	
C3	−0.10761 (10)	0.32364 (13)	0.13788 (6)	0.0161 (3)	
C4	0.08227 (10)	0.47383 (13)	0.11800 (7)	0.0175 (3)	
C5	0.11061 (9)	0.19004 (12)	0.17374 (6)	0.0153 (3)	
H5A	0.1228	0.1152	0.1708	0.018*	
H5B	0.1595	0.2284	0.1783	0.018*	
C6	0.05746 (10)	0.20852 (12)	0.22068 (6)	0.0163 (3)	
H6A	0.0865	0.1962	0.2528	0.020*	
H6B	0.0136	0.1595	0.2196	0.020*	
C7	0.14297 (9)	0.24008 (12)	0.05972 (7)	0.0149 (3)	
C8	0.20274 (10)	0.16476 (14)	0.06237 (7)	0.0206 (3)	
H8A	0.2028	0.1146	0.0890	0.025*	
C9	0.26242 (11)	0.16466 (16)	0.02509 (8)	0.0259 (4)	
H9A	0.3026	0.1149	0.0270	0.031*	
C10	0.26182 (11)	0.23863 (16)	−0.01474 (8)	0.0275 (4)	
H10A	0.3015	0.2381	−0.0397	0.033*	
C11	0.20263 (13)	0.31346 (16)	−0.01777 (8)	0.0293 (4)	
H11A	0.2025	0.3629	−0.0448	0.035*	
C12	0.14324 (11)	0.31449 (14)	0.01975 (7)	0.0231 (4)	
H12A	0.1037	0.3652	0.0180	0.028*	
C13	−0.00267 (9)	0.11792 (13)	0.08554 (7)	0.0152 (3)	
C14	0.00004 (11)	0.01848 (14)	0.10873 (7)	0.0221 (4)	
H14A	0.0320	0.0068	0.1377	0.027*	
C15	−0.04515 (12)	−0.06359 (14)	0.08852 (8)	0.0259 (4)	
H15A	−0.0436	−0.1300	0.1042	0.031*	
C16	−0.09245 (11)	−0.04732 (14)	0.04528 (8)	0.0238 (4)	
H16A	−0.1223	−0.1027	0.0319	0.029*	
C17	−0.09518 (11)	0.05155 (14)	0.02200 (8)	0.0234 (4)	
H17A	−0.1270	0.0627	−0.0071	0.028*	
C18	−0.05043 (11)	0.13430 (13)	0.04200 (7)	0.0212 (3)	
H18A	−0.0524	0.2007	0.0263	0.025*	
C19	−0.06385 (9)	0.35132 (12)	0.27216 (6)	0.0142 (3)	
C20	−0.05732 (10)	0.28751 (13)	0.31664 (7)	0.0184 (3)	
H20A	−0.0130	0.2454	0.3212	0.022*	
C21	−0.11640 (11)	0.28637 (14)	0.35403 (7)	0.0224 (4)	
H21A	−0.1116	0.2436	0.3835	0.027*	
C22	−0.18290 (11)	0.34913 (14)	0.34745 (7)	0.0222 (4)	
H22A	−0.2224	0.3487	0.3726	0.027*	
C23	−0.19024 (10)	0.41239 (14)	0.30323 (7)	0.0204 (3)	
H23A	−0.2347	0.4543	0.2988	0.024*	
C24	−0.13112 (10)	0.41316 (13)	0.26559 (7)	0.0172 (3)	
H24A	−0.1365	0.4551	0.2359	0.021*	
C25	0.10030 (9)	0.43394 (13)	0.25556 (7)	0.0160 (3)	
C26	0.14419 (11)	0.39131 (14)	0.29641 (7)	0.0215 (3)	
H26A	0.1367	0.3212	0.3064	0.026*	
C27	0.19926 (11)	0.45346 (15)	0.32230 (8)	0.0260 (4)	
H27A	0.2281	0.4248	0.3498	0.031*	
C28	0.21143 (11)	0.55748 (15)	0.30750 (8)	0.0269 (4)	
H28A	0.2484	0.5986	0.3250	0.032*	
C29	0.16864 (11)	0.60052 (14)	0.26664 (8)	0.0237 (4)	
H29A	0.1771	0.6703	0.2564	0.028*	
C30	0.11286 (10)	0.53888 (13)	0.24094 (7)	0.0183 (3)	
H30B	0.0837	0.5681	0.2137	0.022*	

### Atomic displacement parameters (Å^2^)

**Table:**

	*U*^11^	*U*^22^	*U*^33^	*U*^12^	*U*^13^	*U*^23^
As1	0.01221 (7)	0.01282 (7)	0.01172 (8)	0.00059 (5)	0.00040 (6)	−0.00029 (5)
As2	0.01295 (8)	0.01347 (7)	0.01191 (8)	0.00115 (5)	−0.00018 (6)	−0.00042 (5)
Cr1	0.01258 (12)	0.01213 (11)	0.01236 (12)	0.00032 (8)	−0.00026 (9)	0.00065 (9)
O1	0.0237 (7)	0.0300 (7)	0.0200 (7)	−0.0057 (5)	−0.0055 (5)	0.0078 (5)
O2	0.0222 (6)	0.0174 (6)	0.0300 (7)	0.0035 (5)	0.0000 (6)	−0.0021 (5)
O3	0.0193 (6)	0.0236 (6)	0.0244 (7)	−0.0038 (5)	−0.0008 (5)	0.0022 (5)
O4	0.0232 (7)	0.0283 (7)	0.0300 (7)	−0.0092 (5)	0.0042 (6)	−0.0022 (6)
C1	0.0151 (7)	0.0155 (7)	0.0187 (8)	−0.0023 (5)	0.0004 (6)	0.0015 (6)
C2	0.0164 (8)	0.0186 (7)	0.0158 (8)	−0.0018 (6)	−0.0014 (6)	0.0006 (6)
C3	0.0191 (8)	0.0159 (7)	0.0133 (8)	0.0028 (6)	−0.0016 (6)	0.0013 (6)
C4	0.0210 (8)	0.0164 (7)	0.0149 (8)	0.0013 (6)	0.0006 (6)	−0.0016 (6)
C5	0.0159 (7)	0.0152 (7)	0.0148 (8)	0.0021 (5)	−0.0004 (6)	0.0002 (6)
C6	0.0193 (8)	0.0147 (7)	0.0149 (8)	0.0023 (6)	0.0010 (6)	0.0001 (6)
C7	0.0126 (7)	0.0171 (7)	0.0151 (8)	−0.0022 (5)	0.0017 (6)	−0.0032 (6)
C8	0.0175 (8)	0.0293 (9)	0.0149 (8)	0.0042 (7)	−0.0003 (7)	−0.0004 (7)
C9	0.0156 (8)	0.0412 (11)	0.0208 (9)	0.0045 (7)	0.0015 (7)	−0.0057 (8)
C10	0.0205 (9)	0.0397 (11)	0.0223 (9)	−0.0073 (8)	0.0086 (7)	−0.0067 (8)
C11	0.0371 (11)	0.0269 (9)	0.0240 (10)	−0.0047 (8)	0.0111 (9)	0.0038 (8)
C12	0.0269 (9)	0.0197 (8)	0.0228 (9)	0.0008 (7)	0.0062 (8)	0.0014 (7)
C13	0.0137 (7)	0.0163 (7)	0.0154 (8)	−0.0002 (5)	0.0024 (6)	−0.0032 (6)
C14	0.0278 (9)	0.0219 (8)	0.0166 (8)	−0.0061 (7)	−0.0036 (7)	0.0014 (6)
C15	0.0353 (11)	0.0192 (8)	0.0232 (9)	−0.0078 (7)	−0.0026 (8)	0.0016 (7)
C16	0.0219 (9)	0.0245 (8)	0.0251 (10)	−0.0058 (7)	0.0018 (8)	−0.0078 (7)
C17	0.0217 (9)	0.0248 (8)	0.0237 (9)	0.0022 (7)	−0.0091 (7)	−0.0052 (7)
C18	0.0233 (9)	0.0184 (7)	0.0218 (9)	0.0026 (6)	−0.0057 (7)	−0.0020 (6)
C19	0.0149 (7)	0.0143 (7)	0.0134 (7)	−0.0015 (5)	0.0011 (6)	−0.0019 (5)
C20	0.0199 (8)	0.0155 (7)	0.0197 (8)	0.0018 (6)	0.0002 (7)	−0.0006 (6)
C21	0.0287 (9)	0.0195 (8)	0.0189 (9)	−0.0015 (7)	0.0039 (7)	0.0030 (6)
C22	0.0209 (8)	0.0240 (8)	0.0217 (9)	−0.0039 (7)	0.0078 (7)	−0.0025 (7)
C23	0.0160 (8)	0.0212 (8)	0.0240 (9)	−0.0001 (6)	0.0016 (7)	−0.0011 (7)
C24	0.0166 (8)	0.0179 (7)	0.0171 (8)	0.0009 (6)	−0.0005 (6)	0.0000 (6)
C25	0.0133 (7)	0.0194 (7)	0.0152 (8)	0.0012 (6)	0.0004 (6)	−0.0028 (6)
C26	0.0216 (9)	0.0234 (8)	0.0197 (9)	0.0025 (7)	−0.0030 (7)	0.0001 (7)
C27	0.0202 (9)	0.0329 (10)	0.0247 (10)	0.0072 (7)	−0.0096 (8)	−0.0061 (8)
C28	0.0172 (8)	0.0310 (9)	0.0326 (11)	0.0021 (7)	−0.0057 (8)	−0.0136 (8)
C29	0.0200 (8)	0.0198 (8)	0.0314 (10)	0.0018 (6)	−0.0023 (7)	−0.0075 (7)
C30	0.0166 (8)	0.0184 (7)	0.0198 (9)	0.0026 (6)	−0.0017 (6)	−0.0035 (6)

### Geometric parameters (Å, °)

**Table:**

As1—C7	1.9367 (16)	C13—C18	1.393 (2)
As1—C13	1.9413 (16)	C14—C15	1.390 (2)
As1—C5	1.9634 (16)	C14—H14A	0.9300
As1—Cr1	2.4461 (3)	C15—C16	1.382 (3)
As2—C25	1.9461 (16)	C15—H15A	0.9300
As2—C19	1.9463 (16)	C16—C17	1.383 (3)
As2—C6	1.9704 (16)	C16—H16A	0.9300
As2—Cr1	2.4512 (3)	C17—C18	1.390 (2)
Cr1—C2	1.8457 (17)	C17—H17A	0.9300
Cr1—C1	1.8511 (17)	C18—H18A	0.9300
Cr1—C3	1.8894 (17)	C19—C24	1.396 (2)
Cr1—C4	1.8935 (18)	C19—C20	1.397 (2)
O1—C1	1.155 (2)	C20—C21	1.387 (2)
O2—C2	1.161 (2)	C20—H20A	0.9300
O3—C3	1.146 (2)	C21—C22	1.392 (3)
O4—C4	1.148 (2)	C21—H21A	0.9300
C5—C6	1.520 (2)	C22—C23	1.389 (3)
C5—H5A	0.9700	C22—H22A	0.9300
C5—H5B	0.9700	C23—C24	1.392 (2)
C6—H6A	0.9700	C23—H23A	0.9300
C6—H6B	0.9700	C24—H24A	0.9300
C7—C12	1.387 (2)	C25—C26	1.392 (2)
C7—C8	1.394 (2)	C25—C30	1.392 (2)
C8—C9	1.393 (3)	C26—C27	1.390 (3)
C8—H8A	0.9300	C26—H26A	0.9300
C9—C10	1.381 (3)	C27—C28	1.382 (3)
C9—H9A	0.9300	C27—H27A	0.9300
C10—C11	1.383 (3)	C28—C29	1.384 (3)
C10—H10A	0.9300	C28—H28A	0.9300
C11—C12	1.393 (3)	C29—C30	1.392 (2)
C11—H11A	0.9300	C29—H29A	0.9300
C12—H12A	0.9300	C30—H30B	0.9300
C13—C14	1.389 (2)		
			
C7—As1—C13	101.03 (7)	C7—C12—C11	120.17 (17)
C7—As1—C5	102.03 (7)	C7—C12—H12A	119.9
C13—As1—C5	105.23 (7)	C11—C12—H12A	119.9
C7—As1—Cr1	119.87 (5)	C14—C13—C18	119.62 (16)
C13—As1—Cr1	117.47 (5)	C14—C13—As1	123.77 (13)
C5—As1—Cr1	109.27 (5)	C18—C13—As1	116.60 (12)
C25—As2—C19	101.42 (7)	C13—C14—C15	119.77 (17)
C25—As2—C6	103.18 (7)	C13—C14—H14A	120.1
C19—As2—C6	101.83 (7)	C15—C14—H14A	120.1
C25—As2—Cr1	119.27 (5)	C16—C15—C14	120.58 (17)
C19—As2—Cr1	119.97 (5)	C16—C15—H15A	119.7
C6—As2—Cr1	108.69 (5)	C14—C15—H15A	119.7
C2—Cr1—C1	88.38 (7)	C15—C16—C17	119.82 (17)
C2—Cr1—C3	92.23 (7)	C15—C16—H16A	120.1
C1—Cr1—C3	87.53 (7)	C17—C16—H16A	120.1
C2—Cr1—C4	91.07 (7)	C16—C17—C18	120.08 (17)
C1—Cr1—C4	92.09 (7)	C16—C17—H17A	120.0
C3—Cr1—C4	176.66 (7)	C18—C17—H17A	120.0
C2—Cr1—As1	177.11 (5)	C17—C18—C13	120.13 (16)
C1—Cr1—As1	93.38 (5)	C17—C18—H18A	119.9
C3—Cr1—As1	90.13 (5)	C13—C18—H18A	119.9
C4—Cr1—As1	86.58 (5)	C24—C19—C20	119.03 (15)
C2—Cr1—As2	95.95 (5)	C24—C19—As2	119.43 (12)
C1—Cr1—As2	173.10 (5)	C20—C19—As2	121.54 (12)
C3—Cr1—As2	86.94 (5)	C21—C20—C19	120.56 (16)
C4—Cr1—As2	93.20 (5)	C21—C20—H20A	119.7
As1—Cr1—As2	82.513 (9)	C19—C20—H20A	119.7
O1—C1—Cr1	176.05 (15)	C20—C21—C22	120.04 (17)
O2—C2—Cr1	176.30 (15)	C20—C21—H21A	120.0
O3—C3—Cr1	177.18 (15)	C22—C21—H21A	120.0
O4—C4—Cr1	179.09 (16)	C23—C22—C21	119.90 (16)
C6—C5—As1	109.55 (11)	C23—C22—H22A	120.0
C6—C5—H5A	109.8	C21—C22—H22A	120.0
As1—C5—H5A	109.8	C22—C23—C24	120.07 (16)
C6—C5—H5B	109.8	C22—C23—H23A	120.0
As1—C5—H5B	109.8	C24—C23—H23A	120.0
H5A—C5—H5B	108.2	C23—C24—C19	120.39 (16)
C5—C6—As2	109.32 (11)	C23—C24—H24A	119.8
C5—C6—H6A	109.8	C19—C24—H24A	119.8
As2—C6—H6A	109.8	C26—C25—C30	119.10 (16)
C5—C6—H6B	109.8	C26—C25—As2	123.05 (13)
As2—C6—H6B	109.8	C30—C25—As2	117.80 (12)
H6A—C6—H6B	108.3	C27—C26—C25	120.06 (17)
C12—C7—C8	119.66 (16)	C27—C26—H26A	120.0
C12—C7—As1	119.68 (13)	C25—C26—H26A	120.0
C8—C7—As1	120.62 (13)	C28—C27—C26	120.47 (18)
C9—C8—C7	119.99 (17)	C28—C27—H27A	119.8
C9—C8—H8A	120.0	C26—C27—H27A	119.8
C7—C8—H8A	120.0	C27—C28—C29	120.02 (17)
C10—C9—C8	119.87 (18)	C27—C28—H28A	120.0
C10—C9—H9A	120.1	C29—C28—H28A	120.0
C8—C9—H9A	120.1	C28—C29—C30	119.70 (17)
C9—C10—C11	120.54 (18)	C28—C29—H29A	120.1
C9—C10—H10A	119.7	C30—C29—H29A	120.1
C11—C10—H10A	119.7	C29—C30—C25	120.64 (17)
C10—C11—C12	119.76 (18)	C29—C30—H30B	119.7
C10—C11—H11A	120.1	C25—C30—H30B	119.7
C12—C11—H11A	120.1		
			
C7—As1—Cr1—C1	58.18 (8)	C10—C11—C12—C7	−0.7 (3)
C13—As1—Cr1—C1	−65.02 (8)	C7—As1—C13—C14	101.21 (16)
C5—As1—Cr1—C1	175.28 (7)	C5—As1—C13—C14	−4.64 (17)
C7—As1—Cr1—C3	145.71 (8)	Cr1—As1—C13—C14	−126.46 (14)
C13—As1—Cr1—C3	22.52 (8)	C7—As1—C13—C18	−78.32 (14)
C5—As1—Cr1—C3	−97.19 (7)	C5—As1—C13—C18	175.83 (13)
C7—As1—Cr1—C4	−33.72 (8)	Cr1—As1—C13—C18	54.01 (14)
C13—As1—Cr1—C4	−156.91 (8)	C18—C13—C14—C15	−0.3 (3)
C5—As1—Cr1—C4	83.38 (7)	As1—C13—C14—C15	−179.84 (14)
C7—As1—Cr1—As2	−127.40 (6)	C13—C14—C15—C16	0.4 (3)
C13—As1—Cr1—As2	109.41 (6)	C14—C15—C16—C17	−0.3 (3)
C5—As1—Cr1—As2	−10.30 (5)	C15—C16—C17—C18	0.1 (3)
C25—As2—Cr1—C2	−72.21 (7)	C16—C17—C18—C13	0.0 (3)
C19—As2—Cr1—C2	53.61 (7)	C14—C13—C18—C17	0.1 (3)
C6—As2—Cr1—C2	170.04 (7)	As1—C13—C18—C17	179.67 (14)
C25—As2—Cr1—C3	−164.14 (7)	C25—As2—C19—C24	107.98 (13)
C19—As2—Cr1—C3	−38.31 (7)	C6—As2—C19—C24	−145.76 (13)
C6—As2—Cr1—C3	78.11 (7)	Cr1—As2—C19—C24	−25.84 (14)
C25—As2—Cr1—C4	19.20 (7)	C25—As2—C19—C20	−72.24 (14)
C19—As2—Cr1—C4	145.03 (7)	C6—As2—C19—C20	34.03 (15)
C6—As2—Cr1—C4	−98.55 (7)	Cr1—As2—C19—C20	153.95 (11)
C25—As2—Cr1—As1	105.32 (5)	C24—C19—C20—C21	−0.7 (2)
C19—As2—Cr1—As1	−128.85 (5)	As2—C19—C20—C21	179.55 (13)
C7—As1—C5—C6	166.75 (11)	C19—C20—C21—C22	0.0 (3)
C13—As1—C5—C6	−88.13 (12)	C20—C21—C22—C23	0.4 (3)
Cr1—As1—C5—C6	38.86 (11)	C21—C22—C23—C24	−0.1 (3)
As1—C5—C6—As2	−49.63 (13)	C22—C23—C24—C19	−0.6 (3)
C25—As2—C6—C5	−87.01 (12)	C20—C19—C24—C23	1.0 (2)
C19—As2—C6—C5	168.11 (11)	As2—C19—C24—C23	−179.24 (13)
Cr1—As2—C6—C5	40.54 (12)	C19—As2—C25—C26	72.05 (15)
C13—As1—C7—C12	103.78 (14)	C6—As2—C25—C26	−33.15 (16)
C5—As1—C7—C12	−147.84 (14)	Cr1—As2—C25—C26	−153.72 (13)
Cr1—As1—C7—C12	−27.06 (16)	C19—As2—C25—C30	−105.36 (14)
C13—As1—C7—C8	−74.09 (14)	C6—As2—C25—C30	149.44 (13)
C5—As1—C7—C8	34.29 (15)	Cr1—As2—C25—C30	28.87 (15)
Cr1—As1—C7—C8	155.07 (12)	C30—C25—C26—C27	0.5 (3)
C12—C7—C8—C9	0.2 (3)	As2—C25—C26—C27	−176.90 (14)
As1—C7—C8—C9	178.04 (14)	C25—C26—C27—C28	−0.6 (3)
C7—C8—C9—C10	−0.6 (3)	C26—C27—C28—C29	0.0 (3)
C8—C9—C10—C11	0.4 (3)	C27—C28—C29—C30	0.6 (3)
C9—C10—C11—C12	0.2 (3)	C28—C29—C30—C25	−0.7 (3)
C8—C7—C12—C11	0.5 (3)	C26—C25—C30—C29	0.2 (3)
As1—C7—C12—C11	−177.42 (15)	As2—C25—C30—C29	177.68 (13)

### Hydrogen-bond geometry (Å, °)

**Table:**

Cg1 is the centroid of the C7–C12 ring.

**Table:**

*D*—H···*A*	*D*—H	H···*A*	*D*···*A*	*D*—H···*A*
C9—H9A···O1^i^	0.93	2.57	3.345 (2)	141
C16—H16A···Cg1^ii^	0.93	2.60	3.519 (2)	169

Symmetry codes: (i) *x*+1/2, −*y*+1/2, −*z*; (ii) −*x*, −*y*, −*z*.
